# Ethnicity-Specific Normative Models of Quadruple Test as a Screening Test for Down Syndrome

**DOI:** 10.3390/medicina57070651

**Published:** 2021-06-24

**Authors:** Praetip Praikaew, Kuntharee Traisrisilp, Chanane Wanapirak, Ratanaporn Sekararithi, Theera Tongsong

**Affiliations:** Department of Obstetrics and Gynecology, Faculty of Medicine, Chiang Mai University, Chiang Mai 50200, Thailand; praetip.pra@gmail.com (P.P.); cwanapir@gmail.com (C.W.); ratanaporn.se@cmu.ac.th (R.S.); theera.t@cmu.ac.th (T.T.)

**Keywords:** alpha-fetoprotein, Down syndrome, estriol, human gonadotropins, inhibin-A, quadruple test, screening

## Abstract

*Background and Objectives:* To establish normative models for median levels of serum biomarkers of the second trimester quad test (alpha-fetoprotein: AFP; free beta-human gonadotropins: hCG; inhibin-A; and unconjugated estriol: uE3) specific to Thai women and to compare multiples of the median (MoMs) derived from ethnicity-specific models and those derived from Caucasian models with ethnic correction. *Materials and Methods:* A cross-sectional study was undertaken in a tertiary, medical teaching center among low-risk pregnant Thai women between 14 and 21 weeks of gestation to measure the levels of the four serum biomarkers. The measured values of each biomarker were analyzed using the multivariable factorial polynomial technique for quantile regression as a function of gestational age and maternal weight. *Results:* The Thai-specific normative models for the four biomarkers were generated and available for use. The MoMs of all individuals generated from our models were significantly different from conventional (Caucasian) models with ethnic correction (Wilcoxon signed-rank test; *p* < 0.0001 for all biomarkers). The MoMs of AFP and hCG from both methods were in agreement, but those from Thai-specific models were significantly higher. However, those of inhibin-A and uE3 were markedly different and ethnic correction was unlikely to be useful. *Conclusions:* The Thai-specific normative models of the quad test as a function of gestational age and maternal weight were constructed using multivariable factorial polynomial models, better than simple quantile regression or log-linear regression used in earlier decades. The analysis of MoMs supports the use of ethnicity-specific models instead of Caucasian models with ethnic correction.

## 1. Introduction

The second trimester serum screening test for fetal Down syndrome, either triple or quadruple (quad) test, is used worldwide because of its acceptable effectiveness. However, several studies support that ethnic factor or biophysical differences among various population groups can strongly affect serum concentrations of biomarkers [1,2,3]. The serum screening tests are usually less effective when applied to other population groups [1]. The main reason for the discrepancy is likely to be inappropriate normative data used to define the abnormality of tests. By conventional practice, most practitioners use the Caucasian normative models with ethnic correction instead of their own specific models. The serum concentrations of biomarkers of the triple test in pregnant Asian women have been demonstrated to be significantly higher than those in the Caucasian population, even after body weight correction [4,5,6]. These biomarkers mainly belong to the fetus, not the mother. Thus, their serum levels vary with maternal size. The same quantity of biomarkers from the placenta results in various serum concentrations, depending on maternal intravascular volume. Large-sized mothers have low levels because of the dilution effect. Additionally, maternal intravascular volume cannot be perfectly represented by maternal weight. This effect cannot be corrected solely by weight correction. Most serum biomarkers in Asian women are usually higher than those in Western women because of smaller body size. In actual practice, the serum screening applied in the Asian population is based on Caucasian normative models with weight correction and ethnic factor correction, which is different for each biomarker. However, even with the use of ethnic factor correction, the conventional method (Caucasian normative model with ethnic correction) is not as effective as the methods using the models developed from the specific population under consideration [7]. Accordingly, to be more effective in screening, the normative references of median concentrations of the serum biomarkers in normal pregnancy specific to the population under consideration must be firstly developed.

Therefore, ethnicity plays an important role in the occurrence of less effective screening for fetal Down syndrome when using standard reference values derived from another ethnic population group [1]. Accordingly, every population should have its own normative data of these biomarkers specific to its ethnicity. The aims of this study are to create normative reference models in predicting serum concentrations of biomarkers used for the quadruple test (alpha-fetoprotein: AFP; free beta-human gonadotropins: hCG; inhibin-A: Inhibin-A; and unconjugated estriol: uE3) in the Thai population with high reliability using comprehensive regression procedures of modern statistics, and to compare the created models with those derived from the models specific to the Caucasian population with ethnic correction, which is the conventional method used in most parts of the world.

## 2. Patients and Methods

This prospective descriptive study was carried out at a tertiary center (medical teaching school), Chiang Mai University, and 15 network hospitals in the northern part of Thailand between June 2015 and May 2017. The study was ethically approved by the institutional review boards, and the women were enrolled with written informed consent. Pregnant women attending our antenatal care clinic (including the network hospitals) who underwent quadruple (quad) test for fetal Down syndrome screening between 14 and 21 weeks of pregnancy were enrolled. The quad test was free of charge, subsidized by the government as a national policy. The participants met the following inclusion criteria: (1) singleton pregnancy; (2) gestational age of between 14 and 21 weeks, based on sonographic biometry in the first half of pregnancy; (3) Thai ethnicity (immigrants or foreigners were not enrolled); (4) normal pregnancy outcomes. Exclusion criteria were as follows: (1) multifetal pregnancy; (2) underlying medical disease, such as pre-gestational diabetes; (3) fetal chromosomal or structural abnormality; 4) loss to follow-up or unknown final pregnancy outcomes. The demographic data of the participants were prospectively recorded in the project record form, including maternal age, gestational age at the time of blood sampling, parity, ethnicity, body weight, smoking habit, and underlying medical diseases. The participants’ blood samples were collected for measurement of the four serum biomarkers (quad test): AFP, free beta-hCG, inhibin-A and uE3. The obtained blood samples were transferred to the laboratory, and the serum was separated with standard centrifugation. The serum samples were processed, and the four serum biomarkers were measured using the DefiaXpress system (Perkin Elmer, Waltham, MA, USA). All laboratory assays and measurements were run in batches to reduce intra-assay variations. The actual serum concentrations of the four biomarkers were then converted to multiples of the median (MoMs), using Caucasian reference models with body weight adjustment [8] and ethnic factor (Asia) correction, which was automatically computed by the built-in software. The combined risk of fetal Down syndrome was also automatically estimated, using the combination of a priori risk (maternal age) and likelihood ratios of serum biomarker MoMs. A combined risk of 1:250 or greater was defined as “high risk” for fetal Down syndrome, and a chromosome study was advised, while in a low-risk case (less than 1:250), it was not suggested.

### Statistical Analysis

The statistical procedures were performed using Stata version 16 (StataCorp. 2019. Stata Statistical Software: Release 16. College Station, TX, USA: StataCorp LLC.). Continuous variables were calculated as mean ± SD or median (IQR), as appropriate, while categorical variables were presented as percentages. The distribution of concentrations of the four serum biomarkers was evaluated by plotting against gestational age of screening. The best-fit models for prediction of median values of the four biomarkers were determined using quantile regression of natural logarithm values of the biomarker levels, against centralized gestational days and centralized body weight, or addition of quadratic terms as appropriate and quantile regression performed through multivariable factorial polynomial technique. The best fit was considered from mean square error (MSE) and pseudo R2. The predicted median values for the four biomarkers specific to a certain gestational age and maternal weight were generated from the regression models and graphically expressed as rainbow contour plots against gestational age and maternal weight. The predicted medians of the biomarkers were calculated based on the generated models, as specific for Thai population. The multiples of the median (MoMs) of each individual were then calculated. The Thai-specific MoMs of each participant were compared with those derived from Caucasian references with ethnic correction, using Wilcoxon signed-rank test.

## 3. Results

During the study period, a total of 6210 singleton pregnancies underwent quad test screens between the gestational age of 14 and 21 weeks. Among them, 491 were excluded because of spontaneous abortion, fetal anomalies, fetal aneuploidy, underlying diseases and incomplete data. The remaining 5719 women were available for analysis. The demographic data of the women are presented in Table 1. All of them were of Thai ethnicity and resided in the northern part of Thailand. The mean maternal weight and mean gestational age at the time of quad screening were 58.4 ± 11.9 kg and 111.5 ± 7.1 days, respectively. It is noteworthy that the rate of smoking was very low among our population.

The distribution of the four biomarkers was not normal. All markers were significantly changed with gestational age and maternal weight. Among various techniques of regression, quantile regression analysis was performed to find the best-fit models using the multivariable factorial polynomial technique, resulting in the best pseudo R2 for all of the four biomarkers. The best-fit models are described below.

Model for predicting AFP:

ln(AFP) = 3.473016 + 0.0215149 × (GA-111.4575975) − 0.6675617 × (ln(BW/100) + 0.5372526622)

or

AFP = e^(3.473016 + 0.0215149 × (GA − 111.4575975) − 0.6675617 × (ln(BW/100) + 0.5372526622))^

Model for predicting free beta-hCG:

ln(hCG) = 2.698273 − 0.0325655 × (GA-111.4575975) − 0.7478521 × (ln(BW/100) + 0.5372526622)

or

hCG = e^(2.698273 − 0.0325655 × (GA − 111.4575975) − 0.7478521 × (ln(BW/100) + 0.5372526622))^

Model for predicting Inhibin A:

ln(Inhibin-A) = 5.274538 − 993.6539 x ((GA/10)^−2^ × ln(GA/10) − 0.0194083493) + 1987.775 x ((GA/10)^−2^ − 0.0080497198) − 0.0065925 x (BW − 58.43612033)

or

Inhibin-A = e^(5.274538 − 993.6539 ((GA/10)^−2 × ln(GA/10) − 0.0194083493) + 1987.775 ((GA/10)^−2 − 0.0080497198) − 0.0065925 (BW − 58.43612033)^

Model for predicting uE3:

ln(uE3) = 1.113706 − 0.005062 × ((GA/10)^−3^ × ln(GA/10) − 3338.86884) + 0.0146575 ((GA/10)^−3^ − 1384.789973) − 0.0054416 (BW − 58.4303412)

or

uE3 = e^(1.113706 − 0.005062 ((GA/10)^3 × ln(GA/10) − 3338.86884) + 0.0146575 × ((GA/10)^3 − 1384.789973) − 0.0054416 (BW − 58.4303412))^

*(GA: gestational age in days; BW: body weight in kg)*

**Figure 1:**
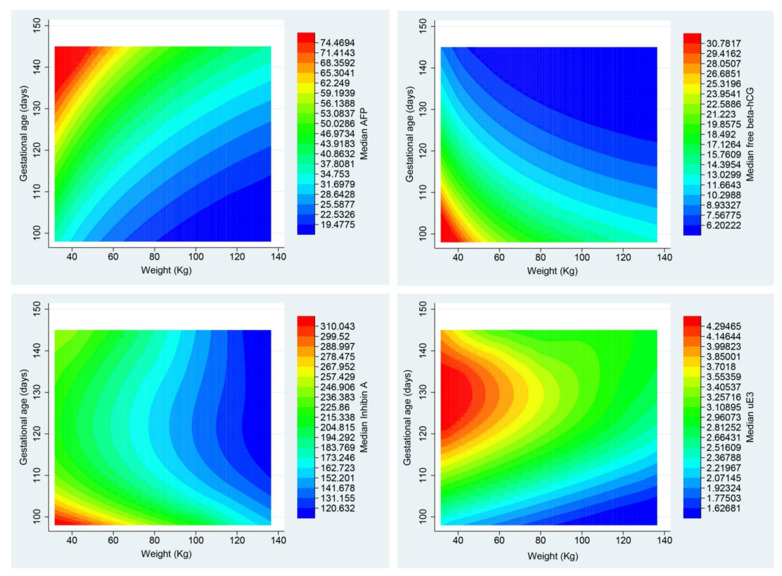
Rainbow contour plots of predicted median alpha-fetoprotein (AFP), free beta-human gonadotropins (beta-hCG), inhibin-A and unconjugated estriol (uE3) as a function of gestational age (days) and maternal weight (kg), based on the generated models in this study.

Based on the generated models, the expected values (median) of the four biomarkers were calculated, specifically to each pregnancy according to maternal weight and gestational age, and then contour plots were constructed, as presented in Figure 1. Note that the levels of all four biomarkers decreased (blue color) with increasing maternal weight, whereas they had different patterns of changes with gestational age. AFP levels increased with increasing gestational age, hCG and inhibin-A levels decreased with increasing gestational age, and uE3 levels initially increased and then decreased.

**Figure 2:**
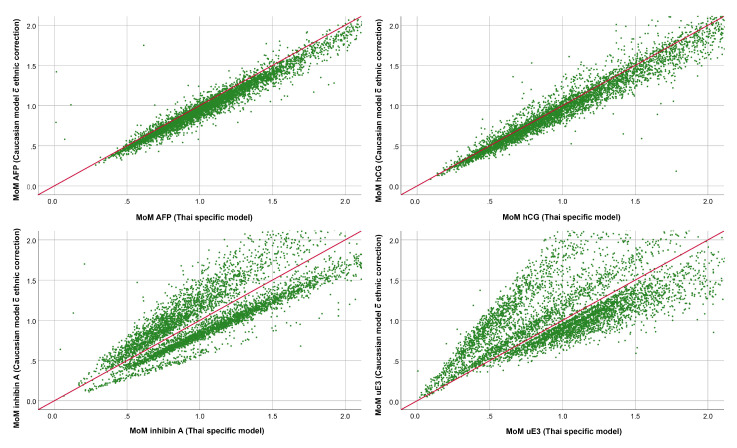
The relationship between the MoMs for AFP, beta-hCG, inhibin-A and uE3, using the Thai model in this study on the X-axis and the Caucasian model with ethnic correction on the Y-axis. The line represents the line of equity or expected MoMs.

According to the generated Thai-specific models described above, the multiples of the median (MoMs) of the four biomarkers specific to each individual were calculated and compared to the MoMs generated from the built-in software, based on the Caucasian reference model with ethnic correction. The comparisons showed that the MoMs of the four biomarkers derived from Thai-specific models and Caucasian-specific models with ethnic correction were significantly different, as presented in Table 2 and Figure 2. It is noteworthy that the MoMs of AFP and hCG of both models were in agreement, though those derived from Thai-specific models were higher. However, the MoMs of inhibin-A and uE3 showed much more discrepancies with greater MoMs.

To compare the consistency of MoMs derived from the two models, the intraclass correlation coefficient (ICC) was analyzed, giving the results as follows: ICCs (95% confidence interval) of MoMs of AFP, hCG, inhibin-A and uE3 are 0.403 (0.371–0.433), 0.443 (0.409–0.474), 0.329 (0.302–0.361), and 0.319 (0.283–0.354), respectively. 

## 4. Discussion

This study established normative models for the median levels of maternal serum biomarkers of the quad test as a function of gestational age and maternal weight. These Thai-specific models are theoretically more appropriate to apply in our population than conventional normative models with ethnic factor correction. Since the MoMs of the four biomarkers generated by ethnicity-specific models were significantly different from those of conventional methods, Thai-specific models are preferable. These normative models may also be more suitable for other populations in nearby geographical areas or those with close ethnicity, especially in Southeast Asia, than those derived from Caucasian models.

We believe that the ethnicity-specific models would be more appropriate, especially if the MoMs of the four biomarkers derived from both models are very different, as seen in our results. However, if the MoMs from the two methods are not significantly different, the method using Caucasian models with ethnic factor correction would be acceptable.

Since the detection and false positive rates of the quad test are based on the accuracy of assays of the biomarkers and comparisons with the highly reliable normative references or expected normal values of each biomarker, the reliability of normative models is of utmost concern. If the normative models are less reliable in predicting the expected medians for each individual, the MoMs of each biomarker for each individual will certainly affect the risk categorization, which is meant to decide whether there is high or low risk for fetal Down syndrome, likely leading to a lower detection rate (an increase in the number of undetected cases of Down syndrome) or a high false positive rate (an increase in the number of unnecessary invasive diagnoses).

According to Figure 2, the MoMs of AFP and hCG based on Thai-specific models go along with those derived from Caucasian-specific models, though the MoMs derived from Thai-specific models tend to be higher than those of Caucasian models throughout the gestational age range. The findings suggest that the difference is probably caused by inappropriate adjustment with the ethnic factor. If we use the built-in software (Caucasian models) for risk calculation, the ethnic factor used for correction needs to be readjusted. It is noteworthy that the MoMs of inhibin-A and uE3 from both Thai- and Caucasian-specific models showed much more discrepancies and are unlikely to be adjusted with simple ethnic correctors with high reliability. In other words, the built-in ethnic correctors for AFP and hCG need to be readjusted, while ethnic correctors for inhibin-A and uE3 are unlikely to be effective. The discrepancies indicate the two models cannot be properly switched to each other, but do not indicate which one is better. Although the model derived from its own population seems theoretically better, comparison of the effectiveness of the two models in detecting Down syndrome needs to be elucidated in future studies. Nevertheless, since all MoMs from population-specific models are significantly different from those derived from non-specific models, even with ethnic correction, all population groups should establish their own normative models, if possible. The discrepancy could be explained by different models which were used to determine specific medians for each individual. Possibly, the models used in earlier reports might not be the best-fit models because of limitations of statistical software in the past.

Since the levels of serum biomarkers at various points of gestational age and maternal weight were not normally distributed, median regression, instead of mean regression, is more appropriate for this type of data. Actually, we tested several methods of regression, such as quantile regression, log-linear weight-adjusted, centering mean or log-linear regression with weight correction, and the addition to the model of quadratic terms. Among them, multivariable factorial polynomial models gave the highest pseudo R2 for all four biomarkers, though the models are relatively complex or difficult for manual calculation. Nevertheless, in actual practice, these Thai-specific models could be readily developed into a user-friendly application for ease of use or they could be integrated into the software of the machine as the main normative reference models, replacing Caucasian models, without ethnic correction.

### Statistics

In the past, most models of normative data of serum biomarkers were derived from a simple log-linear model of the biomarkers as a function of gestational age with subsequent use of maternal weight correction. However, such models were, theoretically, not the best models to describe the association between serum biomarker levels and multivariables such as gestational age and maternal weight, since not all variables were tested and corrected for the interaction between each other. Their correlations are different among various segments of gestational age ranges. The best way to predict the biomarker levels specific to an individual with a certain body weight and gestational age should be based on the models directly developed for multivariables. The association between placental function, the main source of serum biomarkers, and gestational age is very complex. The best-fit models are usually associated with markedly comprehensive and complex equations, which are impossible to calculate with manual calculation and the simple available software used in earlier decades. Very recently, with the advanced development of statistical software, we can now evaluate a variety of complex models to identify the best-fit one, which is more appropriate for some datasets. We took advantage of this newly available statistical technology by using the multivariable factorial polynomial modeling; quantile regression was performed to find the best-fit models. Additionally, we found that the best-fit models for some biomarkers are very complex, different from most simple models derived in the past and very rarely described elsewhere in the development of normative reference models of serum biomarkers for fetal Down syndrome screening. Accordingly, we believe that the models provided in this study have high reliability and should be strongly considered for application in our population and nearby geographical areas. Nevertheless, regarding these ethnicity-specific models, the true effectiveness in screening still needs to be tested with a larger sample size in future studies or in actual practice. However, from our point of view, normative models reported in the past should be revisited to retest on the same datasets with new modern statistical methods, which could not simply be performed with earlier versions of statistical software. This might contribute to an increase in the effectiveness of screening.

The limitations of this study are: (1) The comparison of the MoM differences between the two models was not tested with other sets of pregnant women to determine their reproducibility. (2) The models were not tested for effectiveness in screening for Down syndrome. (3) The impact of smoking was not analyzed because of rarity in our population. The strengths include: (1) The sample size is large enough for the creation of the normative models. (2) Quantile regression analysis was based on multivariable factorial polynomial models, permitting us to identify the models which gave the highest pseudo R2.

## Figures and Tables

**Table 1:** Baseline characteristics of the participants.

Characteristics	Number (%)/Mean ± SD (Range)
Maternal age (years)	28.3 ± 5.4 (13–49)
Maternal weight (kg)	58.4 ± 11.9 (31.6–136.5)
Maternal height (cm)	157.7 ± 5.8 (139–176)
BMI (kg/m^2^)	23.3 ± 4.3 (15.0–49.5)
Gestational age at quad test (days)	111.5 ± 7.1 (98–145)
Gestational age at delivery (weeks)	38.4 ± 1.4 (25–42)
Birth weight (grams)	3104 ± 391 (710–5405)
Parity	
Nulliparous	2975 (52.1%)
Parous	2740 (47.9%)
Smoking	
Non-smoking	4610 (99.8%)
Smoking	8 (0.2%)
Socioeconomic status	
Low	4087 (71.5%)
High	1632 (28.5%)
Maternal age group	
Elderly gravida	608 (10.5%)
Reproductive age	5111 (89.4%)

**Table 2:** Comparisons of MoMs derived from Caucasian and Thai references (Wilcoxon signed-rank test).

Serum Markers	Parameter	MoM (Caucasian References)	MoM (Thai References)	*p*-Value
AFP	Mean	1.045912	1.079865	
	SD	1.519614	0.50892	
	Median	0.94000	0.999082	0.011
	IQR	0.450000	0.473266	
hCG	Mean	1.248527	1.354864	
	SD	1.355584	3.293647	
	Median	0.970000	1.000589	<0.001
	IQR	0.800000	0.849828	
Inhibin A	Mean	1.155741	1.139992	
	SD	1.741734	0.679601	
	Median	0.990000	1.000000	<0.001
	IQR	0.583512	0.596400	
uE3	Mean	1.198435	1.073497	
	SD	2.177826	0.871050	
	Median	1.020000	1.000000	<0.001
	IQR	0.533157	0.649109	

## Data Availability

The data of this report are available from the corresponding authors upon request.

## References

[1] Wanapirak C., Piyamongkol W., Sirichotiyakul S., Tongprasert F., Srisupundit K., Luewan S., Traisrisilp K., Jatavan P., Tongsong T. (2019). Fetal Down syndrome screening models for developing countries; Part I: Performance of Maternal Serum Screening. BMC Health Serv. Res..

[2] Wetta L., Biggio J., Owen J. (2011). Use of ethnic-specific medians for Hispanic patients reduces ethnic disparities in multiple marker screening. Prenat. Diagn..

[3] Lee J.H., Park Y., Suh B., Song S.-M., Kwon O.H., Kim J.-H. (2010). Performance Characteristics of the UniCel DxI 800 Immunoassay for the Maternal Serum Quadruple Test, Including Median Values for Each Week of Gestation, in Korean Women. Ann. Lab. Med..

[4] Brooks K., Chik L., O’Brien J.E., Ayoub M., Johnson M.P., Evans M.I. (1999). Variability of adjustments to indices in determining patient risk in biochemical screening. Fetal Diagn Ther..

[5] Bryant-Greenwood P.K., O’Brien J.E., Huang X., Yaron Y., Ayoub M., Johnson M.P., Evans M.I. (1998). Maternal weight differences do not explain ethnic differences in biochemical screening. Fetal Diagn. Ther..

[6] Obrien J., Dvorin E., Drugan A., Johnson M.P., Yaron Y., Evans M.I. (1997). Race-ethnicity-specific variation in multiple-marker biochemical screening: Alpha-fetoprotein, hCG, and Estriol. Obstet. Gynecol..

[7] Lerthiranwong T., Wanapirak C., Sirichotiyakul S., Tongprasert F., Srisupundit K., Luewan S., Tongsong T. (2018). Strong impact of ethnicity on effectiveness of the first trimester maternal serum screen of fetal Down syndrome. J. Matern.-Fetal Neonatal Med..

[8] Wald N.J., Huttly W.J., Hackshaw A.K. (2003). Antenatal screening for Down’s syndrome with the quadruple test. Lancet.

